# MENTAL HEALTH AND TECHNOLOGY UTILIZATION AMONG MEXICAN AMERICAN INFORMAL CAREGIVERS FOR PATIENTS WITH ADRD

**DOI:** 10.1093/geroni/igad104.1596

**Published:** 2023-12-21

**Authors:** Lin Jiang, Adelaido Garcia-Andres, Cassandra Sarmiento, Lubna Nasr, Isis Macias Moreno, Romeo Escobar, Denise Longoria, John Gonzalez

**Affiliations:** The University of Texas Rio Grande Valley, Edinburg, Texas, United States; Universidad Autonoma de Nuevo Leon, San Nicolas de los Garza, Nuevo Leon, Mexico; Teen health center, Galveston, Texas, United States; Knapp UTRGV Family Medicine Residency, Mercedes, Texas, United States; Knapp UTRGV Family Medicine Residency, Mercedes, Texas, United States; The University of Texas Rio Grande Valley, Edinburg, Texas, United States; The University of Texas Rio Grande Valley, Edinburg, Texas, United States; The University of Texas Rio Grande valley, Edinburg, Texas, United States

## Abstract

**Background:**

The number of Latinos with Alzheimer’s disease and related dementia (ADRD) is expected to rise to 1.3 million by 2050. Mexican Americans with ADRD rely heavily on unpaid informal care. The use of information communication technology (ICT) among caregivers is associated with significant improvements in mental health and social support. However, current ICT studies focus mainly on White American caregivers. This study fills the gap. Method: From November 2021 to November 2022, we collected 115 surveys, of which 11 were collected from in-person interviews at a clinic and 104 were collected online. The survey included the UCLA Loneliness Scale version 3, the Center for Epidemiologic Studies Depression (CES-D), and the Online Social Support Scale.

**Results:**

The participants, aged from 18 to 82 and have cared for their family members with ADRD for two years on average. About 70.4% of them use the internet daily. The results indicated that the more time spent on Instagram, YikYak, and first-person shooter online games, the higher depression the participants had. Also, the more first-person shooter online games they use, the higher is their loneliness. Other social media use is not related to loneliness or depression.

**Conclusion:**

This study suggests that it is necessary to explore an appropriate internet-based ICT for Mexican American informal caregivers to reduce their loneliness and depression. Future research needs to examine their feelings and reflections on using internet-based ICT and discern how their caregiver’s role and Latino culture affect the utilization of technology.

